# Supplementary material for: A case-control regression analysis of liver enzymes in obesity-induced metabolic disorders in South Asian females
Source: PLoS One. 2024 Jul 18;19(7):e0303835. doi: 10.1371/journal.pone.0303835 (PMC11257360; doi:10.1371/journal.pone.0303835)
Supplement: S1 File — (PDF) [file pone.0303835.s001.pdf]

PERFORMA

Patient ID: ____________________

Name: ____________________ CNIC: ____________________ Contact No. : ____________

Age: __________ Gender: ☐ Male ☐ Female

Home Address: ____

Resident since: __________________

Occupation: ________________________ Socio-economic status: __________________________

Marital status: ☐ Married ☐ Single ☐ Widow

Married since: ____________________ Kids: _______________

Anthropometric measurements:

Weight (kg): __________ Height (feet): __________ BMI (kg/m^2): _______________

WC (inches): ____________ HC (inches): ____________ Waist-to-hip ratio: _____________

- Blood Pressure (BP): ______________ Pulse Rate: ________ Body Temp.: ________
- Blood Glucose level: __________________ Blood Cholestrol level: __________________

Appetite and Feeding habits:

☐ Oily food (fried) ☐ Spicy food

☐ Normal food ☐ Fat rich diet

☐ Protein rich diet ☐ Carbohydrate rich diet

**Fat rich diet**	**Protein rich Diet**	**Carbohydrate rich diet**
Chocolate	Egg	Wheat
Nuts	Meat	Cereals
Cheese	Sea food	Rice
Bakery items	Yoghurt	Potatoes

Intake of sweet/dessert: __________________

Eating routine and frequency : ____

☐ Tea ☐ Coffee ☐ Milk Sugar in tea/milk: ____________________

Milk usage in tea/coffee: ☐ more ☐ less ☐ normal

Tea → ☐ strong ☐ light Frequency of intake: _______________________

Addictions:

☐ Caffeine ☐ Nicotine ☐ Cigarette ☐ Hukkah ☐ Naswar

☐ Sleeping pills ☐ Anti-depressants ☐ Paan

Medical History:

☐ Diabetic → ☐ Retinopathy ☐ Neuropathy ☐ Nephropathy

☐ Hypertension ☐ Cardiovascular Disorder ☐ Infertility

❖ **Co-morbidity 1: Diabetes:**

☐ Insulin ☐ Tablets Diabetes since: ____________________

Insulin level: __________ Tablets: _______________ Timing of intake: _______________

❖ **Co-morbidity 2: Cardiovascular diseases**

☐ Ischemic Stroke ☐ Atherosclerosis ☐ Atherothrombosis ☐ Hypertension

BP tablets: ☐ Yes ☐ No BP issue since: ____________________

Frequency of intake: ____________________

❖ **Co-morbidity 3: Infertility**

Treatment if any: __________________________ Medicines: _______________________

- Any other medicines: ___________________________________
- Any other disease history: ____

Family History: Diabetes, BP, Asthma

Exclusion Criteria: HIV, HCV, Cancer, Diabetes Type 1, Hepatitis

Consent:

I hereby voluntarily and willingly agree to participate in this study. The purpose of the study has been discussed. The filled in information is true and should be used for research purpose only.

Signature: ___________________________________

Date: ___________________________________
