# Supplementary figures and images for: A case-control regression analysis of liver enzymes in obesity-induced metabolic disorders in South Asian females
Source: PLoS One. 2024 Jul 18;19(7):e0303835. doi: 10.1371/journal.pone.0303835 (PMC11257360; doi:10.1371/journal.pone.0303835)

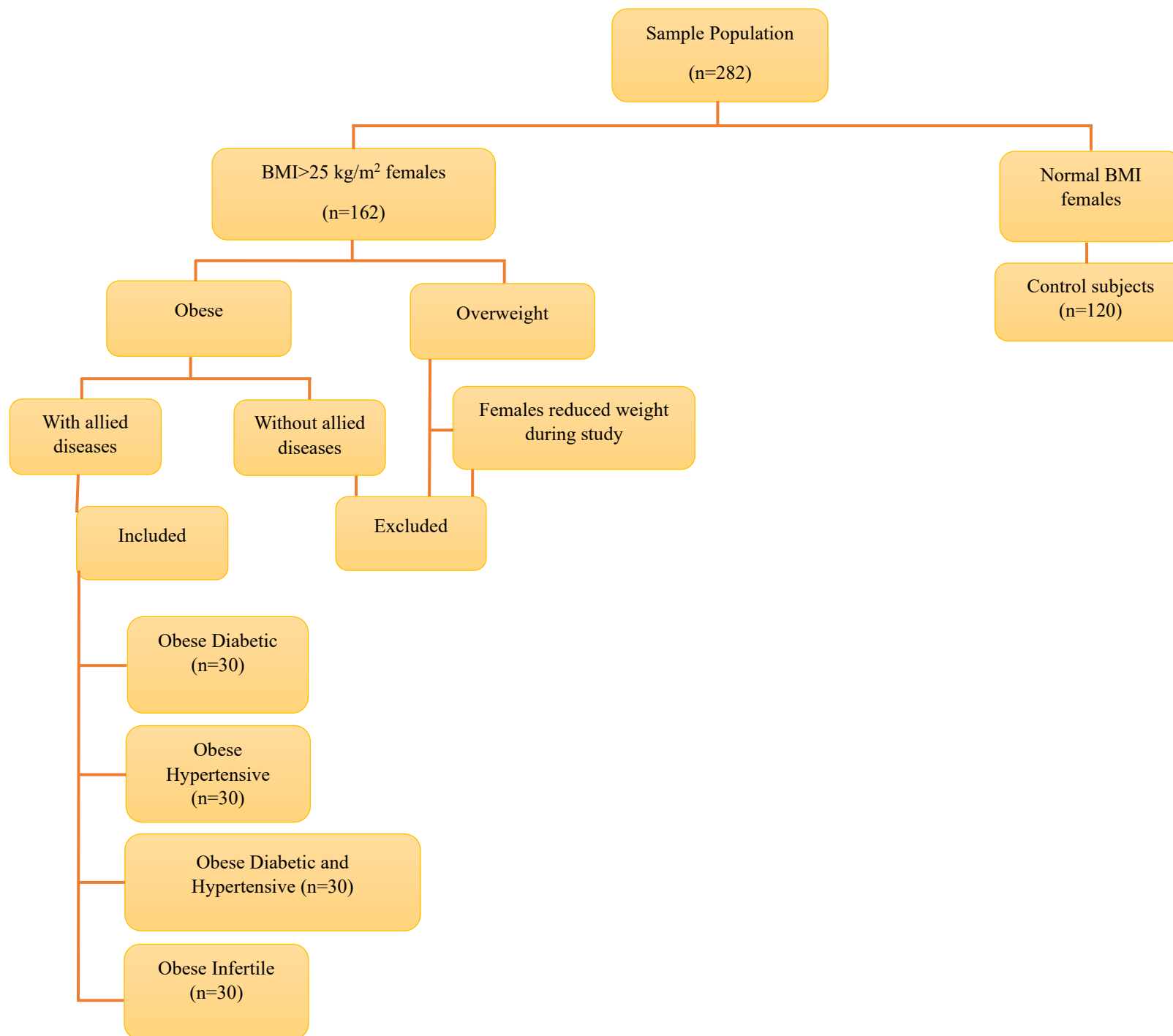

Supplement: S2 File — (PDF) [file pone.0303835.s002.pdf]
