# Supplementary material for: A case-control regression analysis of liver enzymes in obesity-induced metabolic disorders in South Asian females
Source: PLoS One. 2024 Jul 18;19(7):e0303835. doi: 10.1371/journal.pone.0303835 (PMC11257360; doi:10.1371/journal.pone.0303835)
Supplement: S3 File — (PDF) [file pone.0303835.s003.pdf]

Minimal data set of healthy control females																	
**Id**	**Age**	**Weight (kg)**	**Height (inches)**	**BMI kg/m^2**	**BMI Category**	**WC**	**HC**	**WHR**	**SBP (mmHg)**	**DBP (mmHg)**	**Pulse rate**	**Blood Cholesterol level (mg/dl)**	**Blood glucose level (mg/dl)**	**ALT (U/L)**	**AST (U/L)**	**GGT (U/L)**
C1	23	57	5.5	20.9	normal weight	28	36	0.78	110	70	74	145	86	10	24	12
C2	28	67	5.7	23.1	normal weight	32	34	0.94	100	90	70	135	105	12	14	15
C3	24	53	5.4	20.1	normal weight	32	36	0.88	110	70	73	130	100	15	28	15
C4	23	57.3	5	24.7	normal weight	30	37	0.81	120	80	75	148	103	14	23	12
C5	24	46	5.2	18.5	normal weight	26	32	0.81	110	70	76	150	89	16	27	16
C6	23	54	5.1	22.5	normal weight	30	34	0.88	100	70	78	140	92	17	29	15
C7	28	64.5	5.4	24.4	normal weight	31	37	0.84	130	80	75	157	102	13	25	18
C8	31	50	5.2	20.2	normal weight	28	36	0.78	110	70	73	139	100	25	16	15
C9	30	61.7	5.2	24.9	normal weight	30	34	0.88	120	80	75	140	96	21	24	20
C10	24	56	5.6	19.9	normal weight	30	36	0.83	110	70	75	138	105	24	32	11
C11	24	57	5.5	20.9	normal weight	30	37	0.81	110	70	74	146	110	17	30	14
C12	24	63.6	5.3	24.8	normal weight	31	36	0.86	120	80	76	154	120	15	25	59
C13	23	65	5.5	23.8	normal weight	30	37	0.81	110	70	75	133	108	13	30	14
C14	23	64.5	5.4	24.4	normal weight	31	37	0.84	110	70	71	134	109	26	31	59
C15	24	52	5.4	19.7	normal weight	27	33	0.82	110	70	74	142	113	17	23	29

C16	24	53	5.3	20.7	normal weight	27	33	0.82	110	70	75	140	115	14	22	17
C17	26	53	5.1	22.1	normal weight	28	32	0.87	110	70	73	133	99	23	25	13
C18	32	53	5.1	22.1	normal weight	33	35	0.94	120	80	74	152	101	12	18	32
C19	23	48	5.2	19.4	normal weight	27	33	0.82	110	70	75	152	112	20	55	23
C20	24	63	5.4	23.8	normal weight	28	36	0.78	110	70	73	138	98	24	37	17
C21	25	57	5.2	23	normal weight	28	36	0.78	120	80	76	151	115	55	38	36
C22	26	74	5.8	24.8	normal weight	30	34	0.88	120	80	74	147	108	40	35	30
C23	25	54	5.1	22.5	normal weight	26	32	0.81	110	70	71	137	96	35	67	15
C24	24	55	5.5	20.2	normal weight	30	32	0.93	130	80	77	138	120	27	35	20
C25	27	50	5.4	18.9	normal weight	24	28	0.86	110	70	72	136	100	13	24	12
C26	28	53	5.1	22.7	normal weight	28	36	0.78	120	80	71	150	124	17	19	18
C27	25	62	5.5	22.7	normal weight	30	36	0.83	120	80	76	148	103	16	16	20
C28	24	53	5.3	20.7	normal weight	30	34	0.88	110	60	70	149	112	17	23	15
C29	23	48	5.3	18.7	normal weight	27	33	0.82	110	70	72	150	96	58	65	33
C30	26	64	5.5	23	normal weight	30	34	0.88	120	90	74	153	99	24	35	15
C31	24	57	5.5	20.9	normal weight	30	37	0.81	110	70	74	145	110	17	30	14
C32	24	63.6	5.3	24.8	normal weight	31	36	0.86	120	80	76	135	120	15	25	59
C33	23	65	5.5	23.8	normal weight	30	37	0.81	110	70	75	130	108	13	30	14

C34	23	64.5	5.4	24.4	normal weight	31	37	0.84	110	70	71	148	109	26	31	59
C35	24	52	5.4	19.7	normal weight	27	33	0.82	110	70	74	150	113	17	23	29
C36	24	53	5.3	20.7	normal weight	27	33	0.82	110	70	75	140	115	14	22	17
C37	26	53	5.1	22.1	normal weight	28	32	0.87	110	70	73	157	99	23	25	13
C38	32	53	5.1	22.1	normal weight	33	35	0.94	120	80	74	139	101	12	18	32
C39	23	48	5.2	19.4	normal weight	27	33	0.82	110	70	75	140	112	20	55	23
C40	24	63	5.4	23.8	normal weight	28	36	0.78	110	70	73	138	98	24	37	17
C41	23	57	5.5	20.9	normal weight	28	36	0.78	110	70	74	146	86	10	24	12
C42	28	67	5.7	23.1	normal weight	32	34	0.94	100	90	70	154	105	12	14	15
C43	24	53	5.4	20.1	normal weight	32	36	0.88	110	70	73	133	100	15	28	15
C44	23	57.3	5	24.7	normal weight	30	37	0.81	120	80	75	134	103	14	23	12
C45	24	46	5.2	18.5	normal weight	26	32	0.81	110	70	76	142	89	16	27	16
C46	28	53	5.1	22.7	normal weight	28	36	0.78	120	80	71	140	124	17	19	18
C47	25	62	5.5	22.7	normal weight	30	36	0.83	120	80	76	133	103	16	16	20
C48	25	57	5.2	23	normal weight	28	36	0.78	120	80	76	152	115	55	38	36
C49	26	74	5.8	24.8	normal weight	30	34	0.88	120	80	74	152	108	40	35	30
C50	25	54	5.1	22.5	normal weight	26	32	0.81	110	70	71	138	96	35	67	15
C51	24	55	5.5	20.2	normal weight	30	32	0.93	130	80	77	151	120	27	35	20

C52	27	50	5.4	18.9	normal weight	24	28	0.86	110	70	72	147	100	13	24	12
C53	24	53	5.3	20.7	normal weight	30	34	0.88	110	60	70	137	112	17	23	15
C54	23	48	5.3	18.7	normal weight	27	33	0.82	110	70	72	138	96	58	65	33
C55	26	64	5.5	23	normal weight	30	34	0.88	120	90	74	136	99	24	35	15
C56	23	54	5.1	22.5	normal weight	30	34	0.88	100	70	78	150	92	17	29	15
C57	28	64.5	5.4	24.4	normal weight	31	37	0.84	130	80	75	148	102	13	25	18
C58	31	50	5.2	20.2	normal weight	28	36	0.78	110	70	73	149	100	25	16	15
C59	30	61.7	5.2	24.9	normal weight	30	34	0.88	120	80	75	150	96	21	24	20
C60	24	56	5.6	19.9	normal weight	30	36	0.83	110	70	75	153	105	24	32	11
C61	25	62	5.5	22.7	normal weight	30	36	0.83	120	80	76	145	103	16	16	20
C62	24	53	5.3	20.7	normal weight	30	34	0.88	110	60	70	135	112	17	23	15
C63	23	48	5.3	18.7	normal weight	27	33	0.82	110	70	72	130	96	58	65	33
C64	26	64	5.5	23	normal weight	30	34	0.88	120	90	74	148	99	24	35	15
C65	23	57	5.5	20.9	normal weight	28	36	0.78	110	70	74	150	86	10	24	12
C66	28	67	5.7	23.1	normal weight	32	34	0.94	100	90	70	148	105	12	14	15
C67	24	53	5.4	20.1	normal weight	32	36	0.88	110	70	73	149	100	15	28	15
C68	23	57.3	5	24.7	normal weight	30	37	0.81	120	80	75	150	103	14	23	12
C69	25	54	5.1	22.5	normal weight	26	32	0.81	110	70	71	153	96	35	67	15

C70	24	55	5.5	20.2	normal weight	30	32	0.93	130	80	77	130	120	27	35	20
C71	27	50	5.4	18.9	normal weight	24	28	0.86	110	70	72	148	100	13	24	12
C72	28	53	5.1	22.7	normal weight	28	36	0.78	120	80	71	150	124	17	19	18
C73	30	61.7	5.2	24.9	normal weight	30	34	0.88	120	80	75	140	96	21	24	20
C74	24	56	5.6	19.9	normal weight	30	36	0.83	110	70	75	157	105	24	32	11
C75	24	57	5.5	20.9	normal weight	30	37	0.81	110	70	74	139	110	17	30	14
C76	24	63.6	5.3	24.8	normal weight	31	36	0.86	120	80	76	140	120	15	25	59
C77	25	57	5.2	23	normal weight	28	36	0.78	120	80	76	138	115	55	38	36
C78	26	53	5.1	22.1	normal weight	28	32	0.87	110	70	73	146	99	23	25	13
C79	32	53	5.1	22.1	normal weight	33	35	0.94	120	80	74	154	101	12	18	32
C80	23	48	5.2	19.4	normal weight	27	33	0.82	110	70	75	133	112	20	55	23
C81	24	63	5.4	23.8	normal weight	28	36	0.78	110	70	73	134	98	24	37	17
C82	24	46	5.2	18.5	normal weight	26	32	0.81	110	70	76	142	89	16	27	16
C83	23	54	5.1	22.5	normal weight	30	34	0.88	100	70	78	140	92	17	29	15
C84	28	64.5	5.4	24.4	normal weight	31	37	0.84	130	80	75	133	102	13	25	18
C85	31	50	5.2	20.2	normal weight	28	36	0.78	110	70	73	152	100	25	16	15
C86	28	53	5.1	22.7	normal weight	28	36	0.78	120	80	71	152	124	17	19	18
C87	26	74	5.8	24.8	normal weight	30	34	0.88	120	80	74	138	108	40	35	30

C88	23	64.5	5.4	24.4	normal weight	31	37	0.84	110	70	71	151	109	26	31	59
C89	24	52	5.4	19.7	normal weight	27	33	0.82	110	70	74	147	113	17	23	29
C90	24	53	5.3	20.7	normal weight	27	33	0.82	110	70	75	137	115	14	22	17
C91	24	53	5.3	20.7	normal weight	30	34	0.88	110	60	70	138	112	17	23	15
C92	28	53	5.1	22.7	normal weight	28	36	0.78	120	80	71	136	124	17	19	18
C93	25	62	5.5	22.7	normal weight	30	36	0.83	120	80	76	150	103	16	16	20
C94	25	57	5.2	23	normal weight	28	36	0.78	120	80	76	148	115	55	38	36
C95	24	46	5.2	18.5	normal weight	26	32	0.81	110	70	76	149	89	16	27	16
C96	26	64	5.5	23	normal weight	30	34	0.88	120	90	74	150	99	24	35	15
C97	26	74	5.8	24.8	normal weight	30	34	0.88	120	80	74	153	108	40	35	30
C98	25	54	5.1	22.5	normal weight	26	32	0.81	110	70	71	`133	96	35	67	15
C99	24	56	5.6	19.9	normal weight	30	36	0.83	110	70	75	134	105	24	32	11
C100	24	53	5.3	20.7	normal weight	27	33	0.82	110	70	75	146	115	14	22	17
C101	24	57	5.5	20.9	normal weight	30	37	0.81	110	70	74	135	110	17	30	14
C102	24	63.6	5.3	24.8	normal weight	31	36	0.86	120	80	76	150	120	15	25	59
C103	23	65	5.5	23.8	normal weight	30	37	0.81	110	70	75	148	108	13	30	14
C104	23	64.5	5.4	24.4	normal weight	31	37	0.84	110	70	71	149	109	26	31	59
C105	24	52	5.4	19.7	normal weight	27	33	0.82	110	70	74	150	113	17	23	29

C106	30	61.7	5.2	24.9	normal weight	30	34	0.88	120	80	75	153	96	21	24	20
C107	26	53	5.1	22.1	normal weight	28	32	0.87	110	70	73	138	99	23	25	13
C108	32	53	5.1	22.1	normal weight	33	35	0.94	120	80	74	138	101	12	18	32
C109	23	48	5.2	19.4	normal weight	27	33	0.82	110	70	75	151	112	20	55	23
C110	24	63	5.4	23.8	normal weight	28	36	0.78	110	70	73	147	98	24	37	17
C111	23	57.3	5	24.7	normal weight	30	37	0.81	120	80	75	137	103	14	23	12
C112	28	64.5	5.4	24.4	normal weight	31	37	0.84	130	80	75	138	102	13	25	18
C113	31	50	5.2	20.2	normal weight	28	36	0.78	110	70	73	136	100	25	16	15
C114	24	55	5.5	20.2	normal weight	30	32	0.93	130	80	77	150	120	27	35	20
C115	27	50	5.4	18.9	normal weight	24	28	0.86	110	70	72	148	100	13	24	12
C116	28	67	5.7	23.1	normal weight	32	34	0.94	100	90	70	140	105	12	14	15
C117	23	54	5.1	22.5	normal weight	30	34	0.88	100	70	78	157	92	17	29	15
C118	23	57	5.5	20.9	normal weight	28	36	0.78	110	70	74	139	86	10	24	12
C119	23	48	5.3	18.7	normal weight	27	33	0.82	110	70	72	140	96	58	65	33
C120	24	53	5.4	20.1	normal weight	32	36	0.88	110	70	73	138	100	15	28	15

MINIMAL DATA SET OF OBESE HYPERTENSIVE FEMALES

Pt. Id	Age	Weight (kg)	Height (inches)	BMI kg/m^2	BMI Category	WC ('')	HC ('')	WHR	SBP (mmHg)	DBP (mmHg)	Pulse rate	Blood glucose level (mg/dl)	ALT (U/L)	AST (U/L)	GGT (U/L)
1H	33	84	5.2	33.4	class 1 obese	43	43	1	140	100	98	145	10	14	60
2H	55	81	5	34.8	class 1 obese	44	45	0.98	160	80	75	130	68	45	36
3H	45	75	5.2	29.9	overweight	48	45	1.07	170	100	80	120	20	19	17
4H	45	73	5.3	28	overweight	37	41	0.9	140	90	83	110	25	35	28
5H	45	100	5	43	class 3 obese	50	51	0.98	140	90	69	110	64	98	215
6H	43	103	5.2	41	class 3 obese	46	53	0.86	150	90	72	110	20	57	34
7H	41	83	5.3	31.8	class 1 obese	38	42	0.9	130	80	81	100	15	12	29
8H	35	94.2	5	40.5	class 3 obese	48	53	0.91	170	100	79	105	13	13	13
9H	63	80	5.4	30	class 1 obese	50	50	1	180	100	73	110	16	28	44
10H	39	60	5.1 n half	21	normal weight	35	39	0.9	130	80	76	85	19	19	13
11H	40	80	5.2	31.8	class 1 obese	46	48	0.96	140	90	76	130	25	17	30
12H	50	62	5.1	25.8	overweight	44	46	0.96	160	100	101	95	12	15	18
13H	30	100	5.3	38.3	class 2 obese	55	56	0.98	140	90	93	70	11	12	83
14H	60	89.6	5	38.6	class 2 obese	46	48	0.96	130	90	76	99	25	19	22
15H	40	62	5.2	25	overweight	44	46	0.96	140	90	73	100	44	39	107
16H	50	130	5.1	54.1	class 3 obese	46	44	1.05	170	90	73	100	23	25	27
17H	53	64	5.4	24.2	normal weight	37	38	0.97	120	80	73	105	17	11	17

18H	30	78	5.1	32.5	class 1 obese	44	46	0.96	140	90	75	110	11	10	11
19H	42	76	5.1	31.7	class 1 obese	45	45	1	170	110	106	95	16	24	42
20H	47	83	5	35.7	class 2 obese	42	47	0.89	140	90	67	78	12	16	14
21H	32	73	5.1	30.4	class 1 obese	48	46	1.04	120	80	76	95	40	51	48
22H	40	70	5.1	29.2	overweight	40	40	1	160	110	95	100	13	18	57
23H	43	96	5.2	38.7	class 2 obese	40	48	0.83	150	100	93	105	19	24	49
24H	43	92	5.2	37.1	class 2 obese	51	53	0.96	150	100	96	105	14	16	14
25H	37	64	4.1	29.5	overweight	45	43	1.05	140	90	85	97	19	13	65
26H	61	93	5	40	class 3 obese	49	50	0.98	130	90	78	105	20	24	20
27H	46	75	4.11	33.4	class 1 obese	44	45	0.98	170	100	89	102	19	40	18
28H	42	62	5.3	24.2	normal weight	40	42	0.95	160	90	100	95	35	48	32
29H	35	78	5.2	31.5	class 1 obese	44	44	1	130	90	78	115	35	47	31
30H	34	70	5.1	29.2	overweight	48	48	1	160	100	100	130	46	54	56

**Minimal data set of obese infertile females**																
**Pt. Id**	**Age**	**Weight (kg)**	**Height (inches)**	**BMI kg/m^2**	**BMI Category**	**WC ('')**	**HC ('')**	**WHR**	**SBP (mmHg)**	**DBP (mmHg)**	**Pulse rate**	**Blood Cholesterol level (mg/dl)**	**Blood glucose level (mg/dl)**	**ALT (U/L)**	**AST (U/L)**	**GGT (U/L)**
1I	32	75	5.7	24.8	normal weight	34	38	0.89	130	80	72	183	98	23	16	16
2I	37	63	5	27	overweight	38	41	0.93	110	70	75	140	100	32	15	99
3I	25	66	5.1	28	overweight	40	42	0.95	110	70	73	110	100	17	18	19
4I	33	73	5.1	30.2	class 1 obese	42	43	0.98	110	70	74	100	110	14	32	18
5I	27	82.2	5.3	31.5	class 1 obese	43	45	31.5	120	80	74	120	100	25	18	25

6I	41	67	5.2	27	overweight	36	40	0.9	100	70	73	140	90	11	16	19
7I	30	66	5.4	24.5	normal weight	38	39	0.97	110	70	72	130	95	16	12	12
8I	24	67	5.3	26.1	overweight	36	39	0.92	110	70	72	135	95	11	19	13
9I	26	77.6	5.1	32.3	class 1 obese	46	47	0.98	130	80	75	140	104	18	15	10
10I	30	75	5.3	28.7	overweight	41	43	0.96	130	80	73	135	92	27	37	16
11I	27	106.5	5.7	35.3	class 2 obese	45	47	0.96	110	70	75	140	98	26	22	14
12I	35	68	4.11	30.3	class 1 obese	39	45	0.87	130	80	73	135	99	26	16	21
13I	20	84.8	5.1	35	class 2 obese	45	46	0.98	110	70	76	145	137	15	10	12
14I	30	96	5.1	39.7	class 2 obese	50	52	0.96	120	80	72	148	100	25	22	10
15I	24	80	5.1	33.1	class 1 obese	43	44	0.98	130	90	73	138	105	37	16	10
16I	26	73.4	5	31.6	class 1 obese	39	44	0.87	140	90	75	140	110	13	29	15
17I	35	71	5 & half	29.6	overweight	35	42	0.83	130	90	75	200	108	38	31	22
18I	39	123.5	5.4	45.6	class 3 obese	46	57	0.81	130	90	73	125	100	22	10	17
19I	28	58.8	5.3	22.5	normal weight	33	38	0.87	110	70	73	135	110	31	22	19
20I	24	67.4	5.1	27.9	overweight	40	40	1	110	70	75	140	105	24	17	26
21I	32	66.4	5.3	25.4	overweight	39	40	0.98	130	70	73	150	100	15	12	143
22I	23	67.6	5.1	28	overweight	38	39	0.97	120	80	77	160	109	23	23	61
23I	26	75.4	5	32.5	class 1 obese	39	42	0.93	110	70	73	135	99	15	12	12
24I	36	97.4	5.3	37.3	class 2 obese	49	49	1	110	70	73	130	112	18	10	14
25I	31	55	5.2	21.9	normal weight	32	38	0.84	110	70	72	140	80	16	24	13
26I	28	66	5.1	27.3	overweight	36	41	0.88	130	80	75	150	88	15	17	44
27I	30	63	5.2	25	overweight	34	41	0.83	110	70	72	150	80	16	31	18
28I	23	57.8	5.1	24	normal weight	36	39	0.92	110	70	73	154	99	44	50	16
29I	30	75	4.1	34.6	class 1 obese	42	45	0.93	110	70	73	152	105	25	21	36
30I	26	91.6	5.3	35	class 2 obese	47	47	1	110	70	73	140	80	26	21	19
31I	33	73	5.1	30.2	class 1 obese	42	43	0.98	110	70	74	135	110	14	32	18
32I	27	82.2	5.3	31.5	class 1 obese	43	45	31.5	120	80	74	145	100	25	18	25
33I	26	77.6	5.1	32.3	class 1 obese	46	47	0.98	130	80	75	128	104	18	15	10
34I	27	106.5	5.7	35.3	class 2 obese	45	47	0.96	110	70	75	150	98	26	22	14
35I	24	80	5.1	33.1	class 1 obese	43	44	0.98	130	90	73	155	105	37	16	10
36I	26	73.4	5	31.6	class 1 obese	39	44	0.87	140	90	75	128	110	13	29	15
37I	39	123.5	5.4	45.6	class 3 obese	46	57	0.81	130	90	73	145	100	22	10	17

38I	26	75.4	5	32.5	class 1 obese	39	42	0.93	110	70	73	160	99	15	12	12
39I	30	75	4.1	34.6	class 1 obese	42	45	0.93	110	70	73	140	105	25	21	36
40I	27	91.6	5.3	35	class 2 obese	46	46	1	110	70	73	146	80	26	21	19
41I	33	73	5.1	30.2	class 1 obese	42	43	0.98	110	70	74	130	110	14	32	18
42I	26	77.6	5.1	32.3	class 1 obese	46	47	0.98	130	80	75	142	104	18	15	10
43I	20	84.8	5.1	35	class 2 obese	45	46	0.98	110	70	76	154	137	15	10	12
44I	26	73.4	5	31.6	class 1 obese	39	44	0.87	140	90	75	135	110	13	29	15
45I	39	123.5	5.4	45.6	class 3 obese	46	57	0.81	130	90	73	160	100	22	10	17
46I	36	97.4	5.3	37.3	class 2 obese	48	48	1	120	80	73	130	112	18	10	14

Minimal data set of obese diabetic and hypertensive females

Pt. Id	Age	Weight (kg)	Height	BMI kg/m^2	BMI Category	WC ('')	HC ('')	WHR	SBP (mmHg)	DBP (mmHg)	Pulse rate	Blood Cholesterol level (mg/dl)	Blood glucose level (mg/dl)	ALT (U/L)	AST (U/L)	GGT (U/L)
1D+H	45	72	5.2	29	overweight	46	44	1.05	190	100	91	90	222	60	90	34
2D+H	40	79	4.11	50.3	class 3 obese	47	46	1.02	150	100	106	160	382	19	23	27
3D+H	45	94	5.5	33.4	class 1 obese	46	46	1	150	70	80	140	160	46	56	48
4D+H	57	88	5.2	35	class 2 obese	48	45	1.07	160	100	91	135	260	40	30	30
5D+H	52	70	5	30.1	class 1 obese	47	41	1.15	150	90	83	155	427	49	66	78
6I+H	43	77	4.11	34.3	class 1 obese	47	44	1.07	150	110	72	95	100	32	25	27
7H+D	50	99	5.3	37.9	class 2 obese	47	50	0.94	140	80	75	152	260	29	10	47
8D+H	50	95	5	40.9	class 3 obese	46	49	0.94	130	90	73	210	242	36	30	18
9D+H	46	65	4.11	28.9	overweight	45	43	1.04	130	90	75	160	221	77	42	108
10D+H	50	88	5.1	36.7	class 2 obese	45	43	1.04	140	90	73	200	165	45	49	48

11D+ H	40	95	5.2	37.8	class 2 obese	54	53	1.02	130	90	73	200	245	17	19	39
12D+ H	42	99	5.3	37.9	class 2 obese	47	50	0.94	140	90	75	187	321	51	21	12
13D+ H	50	65	5.2	26	overweigh t	45	42	1.07	120	80	80	180	214	41	60	26
14D+ H	40	102	5.2	41.1	class 3 obese	50	52	0.96	150	90	73	210	151	25	35	21
15D+ H	58	70	5.1	29.2	overweigh t	44	42	1.05	210	110	73	200	295	40	33	32
16D+ H	60	90	5.1	37.5	class 2 obese	49	46	1.07	150	90	81	185	170	59	79	21
17D+ H	47	82	5.2	33.1	class 1 obese	44	48	0.92	140	90	76	200	210	23	35	41
18D+ H	50	85	4.1	39.2	class 2 obese	50	48	1.04	140	90	75	185	252	25	30	22
19D+ H	60	76	4.9	36.3	class 2 obese	44	45	0.98	130	90	88	190	250	15	21	13
20D+ H	59	73	5	31.4	class 1 obese	43	42	1.02	130	90	73	195	290	18	26	19
21D+ H	60	80	5.1	33.3	class 1 obese	46	49	0.94	130	90	73	205	222	16	10	30
22D+ H	50	70	5	30.1	class 1 obese	41	41	1	140	90	98	206	242	24	107	66
23D+ H	59	95	5.6	33.8	class 1 obese	48	46	1.04	130	80	93	190	221	22	25	138
24D+ H	60	82	5.2	33.1	class 1 obese	44	41	1.07	150	90	82	205	321	22	29	29
25D+ H	65	62	4.7	31.8	class 1 obese	50	46	1.09	150	100	109	215	139	45	51	16
26D+ H	57	72	5.2	29	overweigh t	45	48	0.94	130	80	76	208	321	16	19	18
27D+ H	60	95	5.1	39.6	class 2 obese	51	50	1.02	140	90	101	230	321	46	35	50
28D+ H	55	75	4.1	34.6	class 1 obese	45	42	1.07	130	90	76	300	135	17	22	55
29D+ H	40	105	5 & half	42	class 3 obese	52	56	0.93	170	90	80	198	192	30	25	37

30D+H	56	65	5.2	26.2	overweight	41	39	1.05	140	90	78	218	221	17	17	34
31D+H	40	79	4.11	50.3	class 3 obese	47	46	1.02	150	100	106	200	382	19	23	27
32D+H	42	99	5.3	37.9	class 2 obese	47	50	0.94	140	90	75	210	321	51	21	12
33D+H	50	99	5.3	37.9	class 2 obese	47	50	0.94	140	80	75	208	260	29	10	47
34D+H	47	82	5.2	33.1	class 1 obese	44	48	0.92	140	90	76	190	210	23	35	41
35D+H	59	95	5.6	33.8	class 1 obese	48	46	1.04	130	80	93	178	221	22	25	138
36D+H	41	102	5.2	41.1	class 3 obese	50	52	0.96	150	90	73	205	151	25	35	21

Minimal data set of obese diabetic females

**Pt. Id**	**Age**	**Weight (kg)**	**Height**	**BMI kg/m²**	**BMI Category**	**WC ('')**	**HC ('')**	**WHR**	**SBP (mmHg)**	**DBP (mmHg)**	**Pulse rate**	**Blood Cholesterol level (mg/dl)**	**Blood glucose level (mg/dl)**	**ALT (U/L)**	**AST (U/L)**	**GGT (U/L)**
1D	30	85	5.2	34.3	class 1 obese	44	46	0.956	120	80	96	124	142	22	18	22
2D	30	112	5.4	41.3	class 3 obese	52	53	0.98	140	90	96	106	281	18	15	48
3D	55	58	4.8	28.7	overweight	40	38	1.05	130	80	80	170	126	14	30	45
4D	45	54	4.1	34.9	class 1 obese	41	42	0.98	100	70	83	135	173	47	60	40
5D	59	62	4.9	29.6	overweight	40	38	1.05	120	80	73	200	350	21	30	104
6D	60	91	5	39.2	class 2 obese	55	52	1.06	130	80	75	186	172	33	38	17
7D	50	77	5	33.2	class 1 obese	49	47	1.04	120	80	74	174	160	21	30	67
8D	52	85	5	36.6	class 2 obese	41	48	0.85	120	80	73	160	180	45	20	27

9D	45	59	4.1	27.2	overweight	45	44	1.02	120	80	72	152	131	24	12	38
10 D	46	60	4.1	27.6	overweight	45	44	1.02	120	80	74	155	312	17	16	36
11 D	40	78	5.1	32.5	class 1 obese	46	43	1.07	120	80	72	160	197	25	35	77
12 D	58	63	4.1	29	overweight	40	41	0.98	130	80	74	180	295	35	28	39
13 D	45	65	5	28	overweight	42	47	0.89	120	80	73	190	212	33	27	22
14 D	50	95	5	40.9	class 3 obese	42	49	0.86	130	80	73	212	257	24	30	30
15 D	60	90	4.1	41.5	class 3 obese	45	46	0.98	120	80	73	170	320	14	27	39
16 D	37	56	4.8	27.7	overweight	36	42	0.86	120	80	72	175	211	16	35	31
17 D	37	53	5	22.8	normal weight	33	37	0.89	120	80	72	170	140	45	48	51
18 D	53	62	4.11	27.6	overweight	44	44	1	120	80	73	198	321	17	36	48
19 D	58	63	4.1	29	overweight	40	40	1	110	70	73	215	295	20	36	59
20 D	37	80	5 & half	34.2	class 1 obese	44	41	1.07	120	80	73	185	141	51	40	52
21 D	50	87	5 & half	37.4	class 2 obese	48	48	1	120	80	73	210	231	42	30	43
22 D	60	88	5.1	36.7	class 2 obese	50	48	1.04	140	90	91	200	221	38	38	63
23 D	53	78	5	33.6	class 1 obese	44	41	1.07	120	80	75	180	160	38	34	34
24 D	57	70	5	30	class 1 obese	41	39	1.05	130	80	75	186	242	64	45	23
25 D	43	69	5.2	27.8	overweight	40	40	1	120	80	74	195	195	21	22	32
26 D	53	61	5	26.3	overweight	42	40	1.05	150	90	71	200	251	32	84	73
27 D	37	62	5.1	25.8	overweight	39	41	0.95	120	80	73	170	355	43	27	21

28 D	50	92	4.1	42.4	class 3 obese	46	48	0.96	140	90	98	180	365	90	134	28
29 D	60	67	4.1	30.9	class 1 obese	42	38	1.11	130	90	80	175	251	59	19	16
30 D	57	78	5	33.6	class 1 obese	46	45	1.02	120	90	75	176	292	13	18	30
31 D	30	112	5.4	41.3	class 3 obese	52	53	0.98	140	90	96	200	281	18	15	48
32 D	52	85	5	36.6	class 2 obese	41	48	0.85	120	80	73	186	180	45	20	27
33 D	40	78	5.1	32.5	class 1 obese	46	43	1.07	120	80	72	174	197	25	35	77
34 D	60	88	5.1	36.7	class 2 obese	50	48	1.04	140	90	91	173	221	38	38	63
35 D	57	70	5	30	class 1 obese	41	39	1.05	130	80	75	182	242	64	45	23
36 D	60	67	4.1	30.9	class 1 obese	42	38	1.11	130	90	80	198	251	59	19	16
37 D	30	85	5.2	34.3	class 1 obese	44	46	0.956	120	80	96	215	142	22	18	22
38 D	50	95	5	40.9	class 3 obese	42	49	0.86	130	80	73	185	257	24	30	30
39 D	60	90	4.1	41.5	class 3 obese	45	46	0.98	120	80	73	172	320	14	27	39
40 D	45	54	4.1	34.9	class 1 obese	41	42	0.98	100	70	83	165	173	47	60	40
41 D	53	78	5	33.6	class 1 obese	44	41	1.07	120	80	75	180	160	38	34	34
42 D	60	67	4.1	30.9	class 1 obese	42	38	1.11	130	90	80	182	251	59	19	16
