# Supplementary material for: A case-control regression analysis of liver enzymes in obesity-induced metabolic disorders in South Asian females
Source: PLoS One. 2024 Jul 18;19(7):e0303835. doi: 10.1371/journal.pone.0303835 (PMC11257360; doi:10.1371/journal.pone.0303835)
Supplement: S4 File — (PDF) [file pone.0303835.s004.pdf]

Regression analysis equation for obese diabetic liver enzymes (Table 2)

$$\hat{y} = 11.068 + 0.145ALT + .059AST - .058GGT$$

Regression analysis equation for obese hypertensive liver enzymes (Table 3)

$$\hat{y} = 16.168 - 0.091ALT + .0139AST - .060GGT$$

Regression analysis equation for obese diabetic and hypertensive liver enzymes (table 4)

$$\hat{y} = 22.28 - 0.259ALT + .002AST + 0.32GGT$$

Regression analysis equation for obese infertile liver enzymes (Table 5)

$$\hat{y} = 9.42 - 0.059ALT + .196AST + 0.029GGT$$

Regression analysis equation for control liver enzymes (Table 6)

$$\hat{y} = 6.42 - 0.78ALT + .91AST + 0.82GGT$$
