# Supplementary material for: A case-control regression analysis of liver enzymes in obesity-induced metabolic disorders in South Asian females
Source: PLoS One. 2024 Jul 18;19(7):e0303835. doi: 10.1371/journal.pone.0303835 (PMC11257360; doi:10.1371/journal.pone.0303835)
Supplement: S5 File — (PDF) [file pone.0303835.s005.pdf]

AST IFCC
Multi-Purpose (MPR) Liquid Reagent

Glenbio
Diagnostics & Research Laboratory Solutions

KIT SPECIFICATIONS:

Cat. No.	*Quantity*	*Reagent*	*Storage*
GL733AS	10 x 15 ml<br>2 x 15 ml	AST IFCC - 1<br>AST IFCC - 2	2 - 8°C
GL743AS	5 x 50 ml<br>1 x 50 ml	AST IFCC - 1<br>AST IFCC - 2	2 - 8°C
GL753AS	5 x 100 ml<br>1 x 100 ml	AST IFCC - 1<br>AST IFCC - 2	2 - 8°C

INTENDED USE:

In Vitro Diagnostic reagent pack for the quantitative determination of Aspartate Aminotransferase (AST) in serum and plasma, based upon the IFCC recommendations, on automated and semi-automated analysers.

SUMMARY AND EXPLANATION: [1]

Aspartate aminotransferase (AST) belongs to a group of enzymes that catalyse the interconversion of amino acids to 2-oxo-acids. The enzyme is largely present in the cytoplasm of cells and highest concentrations are from heart, liver and skeletal muscle tissues. The main clinical uses are in the diagnosis and treatment of myocardial infarction and hepatic diseases. After myocardial infarction, AST level is elevated 6 to 8 hours after the onset of chest pain. It remains elevated for 4 days after which it returns to normal. Five to tenfold elevation of AST are observed in primary metastatic carcinoma of the liver.

Pyridoxyl-5-Phosphate

Pyridoxyl-5-Phosphate functions as a co-enzyme in the aminotransferase reaction. While most patient samples contain endogenous pyridoxyl phosphate the IFCC recommendation specify the addition of the co-enzyme to supplement pyridoxyl-5-phosphate deficient samples. The pyridoxyl-5-phosphate is available as a powder vial from Glenbio Ltd. Cat. No. GL806PP (12 x 50 ml) and GL816PP (6 x 66 ml).

PRINCIPLE OF THE TEST: [1, 2]

The sample is pre-incubated with TRIS (hydroxymethyl) aminomethane (TRIS) buffer solution containing zL-Aspartate, Malate Dehydrogenase (MDH) and Lactate Dehydrogenase (LDH). The reaction is initiated by the addition of NADH and measured kinetically. The rate of NADH consumption is directly proportional to the AST concentration in the patient sample.

$$\text{L-Aspartate} + \alpha\text{-Ketoglutarate} \xrightarrow{AST} \text{Oxaloacetate} + \text{L-Glutamate}$$

$$\text{Oxaloacetate} + \text{NADH} + H^{+} \xrightarrow{MDH} \text{L-Malate} + \text{NAD}^{+}$$

WARNINGS AND PRECAUTIONS:

For In Vitro Diagnostics Use Only - For Professional Use Only
Carefully read instructions for use. Deviations from this procedure may alter performance of the assay.

Components Colour and Appearance:
Reagent 1: Clear colourless liquid.
Reagent 2: Clear pale yellow liquid.
Any significant changes from the above could indicate that the assay might be compromised. Refer to Laboratory's QC program for actions to be taken. In case of serious damage to the bottle and/or cap, resulting in product leakage and/or contamination, do not use the reagent pack and contact your distributor.

Safety precautions:
CAUTION: Take all necessary precautions required when handling laboratory reagents. Contains Sodium Azide. Material Safety Data Sheet is available upon request.

Label Elements:

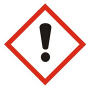

WARNING

H315 Causes skin irritation.
H319 Causes serious eye irritation.

Precautionary Statements:
P280 Wear protective gloves/protective clothing/eye protection/face protection.
P305+P351+P338 IF IN EYES: Rinse cautiously with water for several minutes. Remove contact lenses, if present and easy to do. Continue rinsing.
P332+P313 If skin irritation occurs: Get medical advice/attention.

Handling precautions:
- Do not use components past the expiry date stated on the Bottles.
- Do not Freeze Reagents.
- Do not use components for any purpose other than described in the "Intended Use" section.
- Do not interchange caps among components as contamination may occur and compromise test results.
- Refer to local legal requirements for safe waste disposal.

INSTRUMENTS:

Instrument applications are available upon request.

COMPONENT COMPOSITION:

Component	*Ingredients*	*Concentration in Tests*
Reagent 1	TRIS Buffer pH 7.8	100 mmol/l
	L-Aspartate	300 mmol/l
	MDH	≥ 530 U/l
	LDH	≥ 750 U/l
	STABILISERS & PRESERVATIVES	---
Reagent 2	α-Ketoglutarate	75 mmol/l
	NADH	0.23 mmol/l
	PRESERVATIVES	---

REAGENT PREPARATION AND STABILITY:

Before use, mix reagent by gently inverting each bottle.
If stored and handled properly, unopened components are stable until the expiry date stated on the label

Monoreagent procedure: Add 1 volume of Reagent 2 to 5 Volumes of Reagent 1.
Working reagent is stable 5 weeks at 2-8°C.
Bireagent procedure: Liquid reagent 1 and 2 are ready for use.
Once open, components are stable until expiry date on label if store and handled properly.

TYPE OF SPECIMEN: [1]

Use serum, free of haemolysis, heparin or EDTA plasma as specimen.
It is recommended to follow NCCLS procedures (or similar standardised conditions) regarding specimen handling. Specimen should be collected in an appropriate sample container, with proper specimen identification. Serum/plasma should be separated from cells within 48 hours after collection.
Stability: up to 5 days at 2-8°C[3].

TEST PROCEDURE:

Materials required but not supplied:

Description	*Catalog. No.*	*Description*	*Catalog. No.*
General Chemistry Calibrator	GL983	Photometer	N/A
General Chemistry Control Level 1	GL922	General Laboratory Equipment	N/A
General Chemistry Control Level 2	GL932		

Assay procedure:
Wavelength: λ: 340 nm (Hg 334-Hg 365)
Temperature: 30°C or 37°C
Optical path: 1 cm light path.

MONOREAGENT PROCEDURE:	Blank	Calibrator	Sample
**Working reagent**	1000 µl	1000 µl	1000 µl
**Sample**	----	----	100 µl
**Calibrator**	----	100 µl	----
Gently mix and Incubate at 30°C / 37°C for 1 minute, then measure the change of Optical Density per minute (ΔOD/min) over a further 3 minutes.			

Factor Calculation:
340 nm: U/l = ΔOD/min x 1746   334 nm: U/l = ΔOD/min x 1780   365 nm: U/l = ΔOD/min x 3235

BIREAGENT PROCEDURE:	Blank	Calibrator	Sample
**Reagent 1**	1000 µl	1000 µl	1000 µl
**Sample**	----	----	100 µl
**Calibrator**	----	100 µl	----
Gently mix and Incubate at 37°C for 2 minutes			
**Reagent 2**	200 µl	200 µl	200 µl
Gently mix and Incubate at 37°C for 1 minute, then measure the change of Optical Density per minute (ΔOD/min) over a further 4 minutes			

Factor Calculation:
340 nm: U/l = ΔOD/min x 2063   334 nm: U/l = ΔOD/min x 2103   365 nm: U/l = ΔOD/min x 3823
* The above factors should be validated using General Chemistry Calibrator (AD973).

Enzyme Calibration:
Using recommended Calibrator, calibrate the assay:
- When using a new reagent kit or changing lot number.
- Following preventive maintenance or replacement of a critical part of the photometer used.
- When Quality Controls are out of range.

Quality Control:
All clinical laboratories should establish an Internal Quality Control program. Verify instrument and reagent performance with recommended controls or similar. The values obtained for QC should fall within manufacturer's acceptable ranges or should be established according to the Laboratory's QC program.

Controls should be assayed:
- Prior reporting patient results.
- Following any maintenance procedure on the photometer used.
- At intervals established by the Laboratory QC Programme.

CALCULATION:

340 nm: U/l = ΔOD/min x 1746   334 nm: U/l = ΔOD/min x 1780   365 nm: U/l = ΔOD/min x 3235
(Conversion factor: Qty in µKat/l = Qty in U/l x 0.0167).

EXPECTED VALUES:

IFCC reference method	30°C* U/l	30°C* µkat/l	37°C[4] U/l	37°C[4] µkat/l
Men	Up to 25	Up to 0.42	Up to 37	Up to 0.63
Women	Up to 21	Up to 0.36	Up to 31	Up to 0.53

*Calculated values.   *Temperature conversion factor from 37°C to 30°C: 0.67[4,5].
Each laboratory should establish its own reference range. AST results should always be reviewed with the patient's medical examination and history.

PERFORMANCE CHARACTERISTICS:

Performance results can vary with the instrument used. Data obtained in each individual laboratory may differ from these values.

Linearity:
Linear up to 412 U/l (6.8 µkat/l).
For samples with a higher concentration, dilute 1:1 with 0.9% NaCl (9g/l) and re-assay. Multiply result by 2.

Interfering substances:
Bilirubin (mixed isomers): 12% interference up to 200 µmol/l Bilirubin.
Haemolysis: 12% interference up to 1.25 g/l Haemoglobin.
Lipemia: Less than 10% interference up to 5 g/l Intralipid.

Sensitivity:
The Lowest Detectable Level was estimated at 3 U/l (0.05 µkat/l).

Precision:

Within Run N = 20	*Mean (U/l)*	*SD*	*% CV*	*Between Run N = 20*	*Mean (U/l)*	*SD*	*% CV*
Level 1	35.8	0.624	1.74	Level 1	40	1.09	2.74
Level 2	122.6	0.88	0.72	Level 2	133	2.94	2.22

Method Comparison:
Using 50 samples, a comparison, between this AST test (y) and another commercially available test (x), gave the following results:

y = 1.014x – 0.219	r = 0.999	Sample range: 9 to 355 U/l.

BIBLIOGRAPHY:

1. Bergmeyer HU et. Al. Clin Chem. 1978, 24-58 & Burtis CA, Ashwood ER. Tietz Fund. Of Clin. Chem. 5th ed. 30-54 and 352-390.
2. Tietz NW, ed Clinical Guide to Laboratory Tests, 3rd ed. Philadelphia, Pa: WB Saunders, 1995: 76-77.
3. Fischbach F, Zawta B. Age-dependent Ref Limits of Several Enz in Plasma at Dif Measuring Temp. Klin Lab 1992; 38:555-561.
4. Zawta B, Klein G, Bablok W. Temperature Conversion in Clinical Enzymology Klin Lab 1994; 40:33-42.
5. IFCC Scientific Committee Clin. Chem. Biochem. 1980; 18:521-534.

SYMBOLS:

The following symbols are used in the labelling of Glenbio Ltd. systems:

IVD In Vitro Diagnostics
REF Catalogue No
LOT Batch Code
CONT Content
REAG Reagent
CAL Calibrator
CE Mark - Device comply with the Directives 98/79/EC
Storage temperature
Consult Instruction for Use
Expiry Date (Last day of the month)
Manufactured By

GLENBIO LTD
10 Kilbegs Road, Antrim, Co. Antrim, BT41 4NN
Tel/Fax: +44(0)2879659842
Email: info@glenbio.com
Web: www.glenbio.com

EC REP
GLENBIO IRELAND LTD
17b Fota Business Park, Carrigtwohill, Co. Cork, T45 PK77, Ireland

Revision: 09   Issued on: 20 July 2021
