# Supplementary material for: A case-control regression analysis of liver enzymes in obesity-induced metabolic disorders in South Asian females
Source: PLoS One. 2024 Jul 18;19(7):e0303835. doi: 10.1371/journal.pone.0303835 (PMC11257360; doi:10.1371/journal.pone.0303835)
Supplement: S6 File — (PDF) [file pone.0303835.s006.pdf]

Glenbio
Diagnostics & Research Laboratory Solutions

γ - GT
Multi-Purpose (MPR)
Liquid Reagent

KIT SPECIFICATIONS:

Cat. No.	Quantity	Reagent	Storage
GL705GT	10 x 15 ml	GAMMA GT - 1	2-8°C
	2 x 15 ml	GAMMA GT - 2	
GL715GT	5 x 50 ml	GAMMA GT - 1	2-8°C
	1 x 50 ml	GAMMA GT - 2	

INTENDED USE:

In Vitro Diagnostic reagent pack for the quantitative determination of γ-Glutamyl Transferase (γ-GT) in serum and plasma, (standardised against Szasz) on automated and semi-automated analysers.

SUMMARY AND EXPLANATION: [1]

γ-Glutamyl transferase catalyses the transfer of the γ-Glutamyl group from peptides and compounds that contain this group to some acceptor. The γ-Glutamyl acceptor is the substrate itself. The enzyme is present in all cells except those of muscle. γ-GT present in serum appears to originate primarily from the hepatobilary system and the γ-GT activity is elevated in all forms of liver disease. The measurement of γ-GT is also used in the monitoring of alcohol intake of long-term alcoholic patients.

PRINCIPLE OF THE TEST: [2]

This procedure utilises the water-soluble substrate, Glutamyl Carboxy Nitroanalide. The results correlate with those using the original substrate (Szasz). The increase in absorbance, due to the formation of the p-Nitroanilide, is proportional to the γ-GT concentration in the sample.

$$\text{L-}\gamma\text{-Glutamyl-3-carboxy-4-nitroanilide + glycylglycine} \xrightarrow{\gamma\text{–GT}} \text{L-}\gamma\text{-Glutamyl-glycylglycine + pNitroanilide}$$

WARNINGS AND PRECAUTIONS:

For In Vitro Diagnostics Use Only - For Professional Use Only
Carefully read instructions for use. Deviations from this procedure may alter performance of the assay.

Components Colour and Appearance:
Reagent 1: Clear colourless liquid.
Reagent 2: Pale yellow liquid. (Tends to darken with age but this has no effect on the assay).
Any significant changes could indicate that the assay might be compromised. Refer to Laboratory's QC program for actions to be taken. In case of serious damaged to the bottle and/or cap, resulting in product leakage and/or contamination: do not use the reagent pack and contact your distributor.

Safety precautions:
CAUTION: Take all necessary precautions required when handling laboratory reagents. Contain minute amount of Sodium Azide. Material Safety Data Sheet is available upon request.

Label Elements:

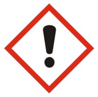

WARNING

H315 - Causes skin irritation.
H319 - Causes serious eye irritation.

Precautionary Statements:
P280 Wear protective gloves/protective clothing/eye protection/face protection.
P305+P351+P338 IF IN EYES: Rinse cautiously with water for several minutes. Remove contact lenses, if present and easy to do. Continue rinsing.
P332+P313 If skin irritation occurs: Get medical advice/attention.

Handling precautions:

- Do not use components past the expiry date stated on the Bottles.
- Do not Freeze Reagents.
- Do not use components for any purpose other than described in the "Intended Use" section.
- Do not interchange caps among components as contamination may occur and compromise test results.
- Refer to local legal requirements for safe waste disposal.

COMPONENT COMPOSITION:

Component	Ingredients	Concentration in Tests
Reagent 1	TRIS Buffer pH 8.25	100 mmol/l
	Glycol Glycine	100 mmol/l
	PRESERVATIVES	---
Reagent 2	Carboxynitroanilide	2.9 mmol/l
	PRESERVATIVES	---

REAGENT PREPARATION AND STABILITY:

Monoreagent procedure: Add 1 volume of Reagent 2 to 5 Volumes of Reagent 1.
Working reagent is stable 3 weeks at 2-8°C.
Bireagent procedure: Liquid reagent 1 and 2 are ready for use.
Before use, mix reagent by gently inverting each bottle.
If stored and handled properly, unopened components are stable until the expiry date stated on the label

INSTRUMENTS:

Instrument applications are available upon request.

TYPE OF SPECIMEN: 1

Serum is the preferred specimen. EDTA plasma may also be used.
Do Not Use Citrate, Oxalate, Fluoride and Heparin.
It is recommended to follow CLSI procedures (or similar standardised conditions) regarding specimen handling. Specimen should be collected in an appropriate sample container, with proper specimen identification. Serum/plasma should be separated from cells as soon as possible after collection.
Stability 3: up to 7 days at 2-8°C.

TEST PROCEDURE:

Materials required but not supplied:

Description	Catalog. No.	Description	Catalog. No.
General Chemistry Calibrator	GL983	Photometer	N/A
General Chemistry Control Level 1	GL922	General Laboratory Equipment	N/A
General Chemistry Control Level 2	GL932		

Assay procedure:
Wavelength: λ: 405 nm
Temperature: 25°C (30°C or 37°C)
Optical path: 1 cm light path.

MONOREAGENT PROCEDURE:	Blank	Calibrator	Sample
**Working reagent**	1000 µl	1000 µl	1000 µl
**Sample**	----	----	100 µl
**Calibrator**	----	100 µl	----
Gently mix and Incubate at 25°C (30°C or 37°C) for 1 minute. Measure the change of Optical Density per minute (ΔOD/min) over the next 3 minutes.			

Factor Calculation: 405 nm: U/l = ΔOD/min x 1158

BIREAGENT PROCEDURE:	Blank	Calibrator	Sample
**Reagent 1**	1000 µl	1000 µl	1000 µl
**Sample**	----	----	100 µl
**Calibrator**	----	100 µl	----
Gently mix and Incubate at 37°C for 3 minutes			
**Reagent 2**	200 µl	200 µl	200 µl
Gently mix and Incubate at 37°C for 2 minutes. Measure the change of Optical Density per minute (ΔOD/min) over the next 4 minutes			

Factor Calculation: 405 nm: U/l = ΔOD/min x 1368
*The above factors should be validated using General Chemistry Calibrator (AD973).

Enzyme Calibration:
Using recommended Calibrator, calibrate the assay:
- When using a new reagent kit or changing lot number.
- Following preventive maintenance or replacement of a critical part of the photometer used.
- When Quality Controls are out of range.

Quality Control:
All clinical laboratories should establish an Internal Quality Control program. Verify instrument and reagent performance with recommended controls or similar. The values obtained for QC should fall within manufacturer's acceptable ranges or should be established according to the Laboratory's QC program.
Controls should be assayed:
- Prior reporting patient results.
- Following any maintenance procedure on the photometer used.
- At intervals established by the Laboratory QC programme.

CALCULATION:

405 nm: U/l = ΔOD/min x 1368
(Conversion factor: Qty in µKat/l = Qty in U/l x 0.0167).

EXPECTED VALUES:

	U/l at 37°C5*	µkat/l at 37°C5*
Men	8 to 61	0.13 to 1.02
Women	5 to 36	0.08 – 0.6

*Conversion factors are available for other temperatures6.

Each laboratory should establish its own reference range. γ-GT results should always be reviewed with the patient's medical examination and history.

PERFORMANCE CHARACTERISTICS:

Performance results can vary with the instrument used. Data obtained in each individual laboratory may differ from these values.

Linearity:
Linear up to 1196 U/l. (20 µKat/l).
For samples with a higher concentration, dilute 1:1 with 0.9% NaCl (9g/l) and re-assay. Multiply result by 2.

Interfering substances:
Bilirubin (mixed isomers): Less than 10% interference up to 600 µmol/l Bilirubin.
Haemolysis: Less than 10% interference up to 5 g/l Haemoglobin.
Lipemia: Less than 10% interference up to 5 g/l Intralipid.

Sensitivity:
The Lowest Detectable Level was estimated at 1 U/l (0.016 µKat/l).

Precision:

Within Run N = 20	Mean (U/l)	SD	% CV	Between Run N = 20	Mean (U/l)	SD	% CV
Level 1	44.4	0.96	2.18	Level 1	44.3	1.44	3.26
Level 2	194.4	1.35	0.69	Level 2	194.3	4.29	2.20

Method Comparison:
Using 50 samples, a comparison, between this γ-GT test (y) and another commercially available test (x), gave the following results:

y = 0.939x + 0.345	r = 0.999	Sample range: 5 to 345 U/l

BIBLIOGRAPHY:

1. Burtis CA, Ashwood ER. Tietz Fund. Of Clin. Chem. 5th ed. 30-54, 370-372 and 984.
2. Szasz G., Clin. Chem., 22 (1976) 2051.
3. Szasz G, Persijn JP, et al. Z Klin Chem Klin Biochem 1974; 12:228.
4. Szasz G. Methods of Enzymatic Analysis. 2nd English ed. New York: Academic Press, Inc. 1974:717.
5. Abicht K et al., Multicenter Evaluation of New Liquid GGT and ALP Reagents with New Reference Standardisation and Determination of Reference Intervals. Clin Chem Lab Med 2001; 39, Special Supplement pp S1-S448, May 201.
6. Zwata B, Klien G, Bablok W. Temperature Conversion in Clinical Enzymology? Klin Lab 1994, 40:33-42.

SYMBOLS:

The following symbols are used in the labelling of Glenbio systems:

IVD In Vitro Diagnostics
REF Catalogue No
LOT Batch Code
CONT Content
REAG Reagent
CAL Calibrator
CE Mark - Device comply with the Directives 98/79/EC
Storage temperature
Reconstitute with
Expiry Date (Last day of the month)
Manufactured By
Biological risk
Consult Instruction for Use

GLENBIO LTD
10 Kilbegs Road, Antrim, Co. Antrim, BT41 4NN
Tel/Fax: +44(0)2879659842
Email: info@glenbio.com
Web: www.glenbio.com

EC REP
GLENBIO IRELAND LTD
17b Fota Business Park, Carrigtwohill, Co. Cork,
T45 PK77, Ireland

Revision: 10 Issued on: 22 July 2021
