# Supplementary material for: A case-control regression analysis of liver enzymes in obesity-induced metabolic disorders in South Asian females
Source: PLoS One. 2024 Jul 18;19(7):e0303835. doi: 10.1371/journal.pone.0303835 (PMC11257360; doi:10.1371/journal.pone.0303835)
Supplement: Attachment — Submitted filename: Response to Reviewers.docx [file pone.0303835.s009.docx]

**Dear Dr. Samiullah Khan**

**Academic Editor**

**PLOS ONE**

All the authors are very grateful for evaluating the manuscript through critical peer review. The reviewers indeed swotted the article with an eagle eye that depicts their competency. They raised such valuable points that greatly altered the study design and its outcome as well. We are much indebted for the reviewers' valuable comments and suggestions that improve the article's quality. The manuscript has been revised in light of reviewers recommendation. A detailed, point-to-point response to the reviewers’ comments is as follows

|  | **Reviewer Comments** | **Response** |
| --- | --- | --- |
|  | **Reviewer 1** |  |
| 1 | To control diabetes or blood pressure, patients may have been treated with drugs. How did you separate their effects on liver enzymes from the effects of obesity on liver enzymes? | It was made sure that patients either did not have any treatment for obesity-related co-morbidities or if treated, they must have shallow potency treatment that could not affect the efficacy of live function. See page 5 lines 107-109 |
| 2 | Not consuming alcohol should be an exclusion criterion | The study population is assumed to not be addicted to alcohol however to resolve the concern it has been mentioned in the manuscript. See page 5 line 106. |
| 3 | Triglyceride, bilirubin and PT-INR - could provide more comprehensive information. Are these data available | Yes, these data are available but are part of another manuscript that is under process, hence cannot be provided. |
| 4 | In daily practice, ultrasound of the upper abdomen and fibro scan of the liver provide valuable information about liver disease. It is even possible that the liver enzymes are normal and the fibro scan shows moderate degrees of fibrosis. Not using liver imaging is one of the limitations of the study and should be mentioned | The study was focused on serology and variations in liver enzyme production due to obesity, therefore, a fibro scan or ultrasound of the upper abdomen was not performed. A fibro scan with mild fibrosis may not fulfil the study objectives. This point could be valid if the study focused on liver echotexture rather than its function. However, the authors appreciated the logic, and they conceived the idea that obesity may cause fibrosis which may be asymptomatic with normal liver enzymes |
|  | **Reviewer 2** | Response |
| 1 | Title: The manuscript title is a critical element that sets the tone for the study. To enhance clarity, it may be beneficial to rephrase the title to specify that the study exclusively focuses on data obtained from female individuals. Additionally, consider including the demographic area of the population, for instance, "South Asian Female Population," to provide a more precise context. | Thanks for bringing this point to our attention. Indeed, it is a very good suggestion. The title has been rephrased as suggested. See title page 1 lines 1-3 |
| 2 | Abstract: I would like to kindly point out that the abstract in the manuscript appears to be incomplete | Acknowledged. The word cirrhosis was omitted from the abstract typographically. Issue resolved. see page 2 line 35 |
| 3 | Results:  Descriptive Titles: I recommend providing more descriptive titles for the ‘Results’ section that directly convey the key observations. This will help readers navigate the content more effectively. | Titles of results sections have been changed that indicate the key observations of results being discussed. |
| 4 | Combining WHR and BMI Results: Given the brevity of the WHR and BMI sections and their shared focus on size-related factors, it could be advantageous to combine these into a single section to streamline the presentation. | The sections have been combined. See title page 6 lines 129-130 |
| 5 | Improving Table Legends: Enhance the clarity of table legends in terms of variable comparisons, particularly in Table 1, and ensure that the context of p-values is clearly defined. If applicable, consider conducting pairwise comparisons to improve data interpretation. | Table 1 legends are described more clearly indicating P=0.05 and pairwise comparisons are indicated in the form of percentage increase or decrease. Table 2 Page # 8 |
|  | Visualization Enhancements: For Figure 1, I suggest utilizing a box plot format with overlaying individual scatter dots, which can provide a clearer representation of the data compared to dynamite plots. | Thank you for your valuable suggestion scatter plot provides a better illustration of data. Figure 1 is updated with individual scatter dots. See Fig 1 |
|  | Incorporate Regression Dot Plots: In addition to tables, it would be beneficial to present regression dot plots, displaying all data points alongside the main results. Additionally, consider including correlation plots with confidence intervals, and if authors find it suitable, these analyses can be integrated into a single plot. | Regression dot plots incorporated as suggested. See Fig. 7.  The correlation plots with confidence intervals cannot be integrated into a single plot, thus presented in a table format. See page no.15 Table 8 |
|  | Clear and Accessible Conclusions: Concluding each results section with a clear and less technical statement summarizing the main findings would greatly benefit the manuscript. This will facilitate a more accessible understanding of the research outcomes. | Done as suggested. |
|  | Supplementary Equations: To streamline the manuscript, it would be better to move the equations in line number 177/185/194/202/209 (e.g., yˆ=x+y+z) from the main text to a supplementary section, citing them where necessary for clarity. | The equations have been moved from the main file to the supplementary file (S8 File). |
|  | Cholesterol Data and Correlations: To provide a comprehensive context, consider including the correlation between cholesterol and obesity. Additionally, clarify whether the authors exclusively measured total cholesterol or if data is available for HDL, LDL, and triglycerides. Correlating this data with obesity would enhance the manuscript's depth. | We appreciate your suggestion; indeed, it has given a new dimension to our manuscript as we have to go through the results once again thoroughly. Correlation between cholesterol and BMI is done as suggested in each respective comorbid group. See Fig. 2-6. Further data about HDL, LDL, and triglycerides cannot be provided now because it is used in another manuscript that is under process. |
