# Supplementary material for: A case-control regression analysis of liver enzymes in obesity-induced metabolic disorders in South Asian females
Source: PLoS One. 2024 Jul 18;19(7):e0303835. doi: 10.1371/journal.pone.0303835 (PMC11257360; doi:10.1371/journal.pone.0303835)
Supplement: Attachment — Submitted filename: Response to Reviewers.docx [file pone.0303835.s010.docx]

**Dear Dr. Samiullah Khan**

**Academic Editor**

**PLOS ONE**

The authors are quite grateful to the Academic Editor for evaluating the work through a critical peer review panel. The selection of reviewers in an article review is crucial and the academic editor did his job very well. The reviewers' keen eye for in-depth analysis demonstrated their proficiency as they thoroughly examined the article. Their insightful observations significantly changed both the study's design and conclusion. We greatly appreciate the perceptive criticism and recommendations from the reviewers, which raise the calibre of the paper. The manuscript has been updated based on the reviewer's and academic editor’s suggestions.

**Response to Journal Requirement and Academic Editor Comments**

To the best of my knowledge, references are complete and correct. Not a single paper is cited that has been retracted. No reference has been added or removed after the peer review process. All the references are up to date and according to the format of Plos One

A detailed, point-to-point response to the reviewers’ comments is as follows

|  | **Reviewer Comments** | **Response** |
| --- | --- | --- |
|  | **Reviewer 2** |  |
| 1 | Please mention the post-hoc tests performed after ANNOVA in the methods and the figure legends. | Added see line 1236-126 |
| 2 | Please mention the number of samples i.e., n=? at appropriate places including figure legends. | Mentioned 171-173 |
| 3 | Please write the key observation as the title of each result instead of writing the method and the statistical test used. For example, result 1 shows the relationship between obesity, diabetes, and hypertension on key health indicators in women. | subtitles of the results have been revised as per the suggestion see lines  176-177  203  239-240 |
| 4 | In Figure 2,3,4,5 & 6: 1. Please include Correlation coefficient (R2) and the P-value on the regression plots. 2. Please mention axis titles. 3. It would be visually appealing to put all these plots in a single page. 4. All the plots should have consistent Y axis limits for enhanced clarity and interpretability. | Revised as per suggestion   1. Correlation coefficient and P-value on the regression plot added 2. Axis titles are mentioned 3. All plots are on a single page 4. It is not possible for all plots to have the same y-axis because each parameter has a different unit value |

We hope that the revised version of the manuscript is appealing enough to satisfy the reviewers and make the understanding of the research better. **We believe that the manuscript is now suitable for publication in PLOS ONE**

Best Regard

Dr. Tamseela Mumtaz (On behalf of all authors)

Assistant Professor, Department of Zoology

Government College Women University, Faisalabad
